# Marcadores de Perfusão Tecidual como Preditores de Desfechos Adversos em Pacientes com Disfunção Ventricular Esquerda Submetidos à Revascularização Miocárdica (Bypass Coronário)

**DOI:** 10.36660/abc.20230247

**Published:** 2024-03-13

**Authors:** Thiana Yamaguti, José Otavio Costa Auler, Luís Alberto Oliveira Dallan, Filomena Regina Barbosa Gomes Galas, Ligia Cristina Câmara Cunha, Marilde de Albuquerque Piccioni

**Affiliations:** 1 Universidade de São Paulo Faculdade de Medicina Instituto do Coração do Hospital das Clínicas São Paulo SP Brasil Instituto do Coração do Hospital das Clínicas da Faculdade de Medicina da Universidade de São Paulo, São Paulo, SP – Brasil

**Keywords:** Cirurgia Torácica, Disfunção Ventricular Esquerda, Biomarcadores

## Abstract

**Fundamento:**

Pacientes submetidos à cirurgia cardíaca podem estar expostos à hipoperfusão tecidual e metabolismo anaeróbico.

**Objetivo:**

Verificar se os biomarcadores de hipoperfusão tecidual têm valor preditivo para permanência prolongada na Unidade de Terapia Intensiva (UTI) em pacientes com disfunção ventricular esquerda submetidos à cirurgia de bypass da artéria coronária.

**Métodos:**

Após aprovação pelo comitê de ética institucional e assinatura do termo de consentimento, 87 pacientes com disfunção ventricular esquerda (fração de ejeção <50%) submetidos à cirurgia de bypass coronário foram incluídos. Biomarcadores hemodinâmicos e metabólicos foram coletados em cinco momentos: após anestesia, ao final da cirurgia, na admissão na UTI, e a seis e 12 horas depois. Uma análise de variância para medidas repetidas seguida de um teste post-hoc de Bonferroni foi usado para variáveis contínuas repetidas (variáveis metabólicas e hemodinâmicas) para determinar diferenças entre os dois grupos ao longo do estudo. O nível de significância adotado foi de 5%.

**Resultados:**

Trinta e oito pacientes (43,7%) que apresentaram desfechos adversos eram mais velhos, apresentaram um Euroscore mais alto (p<0,001), e gradiente venoarterial de CO_2_ (ΔPCO_2_) elevado, analisados 12 horas após a admissão na UTI (p<0,01), enquanto uma concentração de lactato arterial aumentada seis horas após a cirurgia foi um fator preditivo negativo (p<0,01).

**Conclusões:**

EuroSCORE, lactato arterial seis horas após a cirurgia, ΔPCO_2_12 horas após a cirurgia e QRe são preditores independentes de desfechos adversos em pacientes com disfunção ventricular esquerda após cirurgia cardíaca.

## Introdução

Pacientes submetidos à cirurgia cardíaca podem ser expostos à hipoperfusão tecidual e ao metabolismo anaeróbico.^1^ Os fatores envolvidos na hipoxemia celular são hemodiluição, baixo débito cardíaco, e reações inflamatórias após o *bypass* cardiopulmonar (BCP).^2 – 4^ Os biomarcadores de hipoperfusão tecidual, tais como níveis de lactato, gradiente venoarterial de CO_2_ (ΔPCO_2_), quociente respiratório estimado (QRe), e saturação venosa central de oxigênio (SvO2), são aumentados por um débito cardíaco baixo.^3 , 5 – 8^ Os níveis de lactato durante estados de choque estão elevados devido à hipóxia tecidual, metabolismo aumentado da glicose, e *clearance* de lactato reduzido.^1^ O ΔPCO_2_ aumenta com o baixo débito cardíaco ou perfusão inadequada da microcirculação, que significa estagnação do fluxo sanguíneo no compartimento venoso.^6^ Ainda, o QRe (ΔPCO_2_ corrigido pela diferença entre o teor de oxigênio arterial e venoso) mostrou melhor sensibilidade para a detecção do metabolismo anaeróbico em comparação ao ΔPCO_2._^7^

Contudo, a hipoperfusão tecidual poderia estar mascarada pela resposta inflamatória e pelo estado de vasodilatação após o *bypass* .^8 – 11^ Pacientes com disfunção ventricular esquerda submetidos à cirurgia cardíaca são mais propensos a apresentar síndrome de baixo débito cardíaco associada com uma resposta inflamatória sistêmica.^12^ A situação resulta em uma permanência prolongada na Unidade de Terapia Intensiva (UTI) e maiores taxas de mortalidade em comparação a pacientes com função ventricular normal.^13^ A detecção precoce da hipoperfusão tecidual pode predizer desfechos adversos nesse grupo de pacientes que contribuem à internação prolongada na UTI e alta mortalidade. Otimizar o perfil hemodinâmico com base em certos valores de índice cardíaco e no fornecimento de oxigênio às células é a principal regra no manejo pós-operatório. A associação entre aa medida do débito cardíaco e biomarcadores da perfusão tecidual pode definir o suporte com drogas vasoativas e ajuste de volume. A pergunta abordada neste estudo é se os biomarcadores de hipoperfusão tecidual têm um valor preditivo para permanência prolongada na UTI em pacientes com disfunção ventricular submetidos a *bypass* de artéria coronária.

## Métodos

Este é um estudo observacional prospetivo conduzido há mais de dois anos no Instituto do Coração (InCor) do Hospital das Clínicas da Faculdade de Medicina da Universidade de São Paulo. Todos os pacientes assinaram um termo de consentimento, e o comitê de ética local aprovou o estudo (CAPPESQ n. 0517/04).

### Pacientes

Este foi um estudo observacional prospectivo do tipo coorte conduzido durante 24 meses consecutivos. Durante esse período, 183 pacientes com disfunção ventricular esquerda (fração de ejeção < 50%) foram submetidos ao *bypass* coronário e 110 pacientes foram elegíveis para o estudo. Vinte e três pacientes foram excluídos. A Fração de Ejeção do Ventrículo Esquerdo (FEVE) foi medida antes da cirurgia usando ecocardiografia bidimensional. Os critérios de exclusão foram insuficiência renal ( *clearance* de creatinina menor que 40 mL/min/m^2^), disfunção hepática, doenças endócrinas, doença pulmonar, diabetes mellitus não controlada, história de febre ou infecção na semana anterior à cirurgia, e anemia prévia (hemoglobina ≤ 10,0 g/dL).

### Anestesia, cirurgia e manejo do BCP

A anestesia foi induzida com fentanil 3-5 μg/Kg, midazolam 0,05 mg/kg, etomidato 0,2-0,3 mg/kg, relaxamento muscular, e fentanil e sedoflurano para manutenção. O monitoramento padrão para cirurgia cardíaca foi usado, e um cateter foi introduzido na artéria pulmonar para monitoramento hemodinâmico. Uma dose inicial de 500UI de heparina foi administrada para anticoagulação, e o tempo de coagulação ativado foi fixado a 480 segundos. Ao final do BCP, a ação da heparina foi neutralizada pelo cloreto de protamina, na proporção 1:1 da dose de ataque, independentemente da dosagem total de heparina. O fluxo sanguíneo durante o BCP foi estabelecido entre 2,0 e 2,4mL/m^2^ ou de acordo com a pressão arterial média mantida a aproximadamente 60mm Hg. Um oxigenador de membrana foi usado em todos os pacientes, e cardioplegia sanguínea fria intermitente (a cada 10 minutos, anterógrada) foi usada para proteção do miocárdio. Durante o BCP, a temperatura central foi mantida a 32-34^o^C, e os níveis de hematócrito foram entre 22% e 25%.

### Coleta de dados e definições

Os dados demográficos, clínicos e operatórios registrados foram idade (anos), sexo, peso (Kg), Área da Superfície Corporal (ASC, m^2^) Euro SCORE,^14 , 15^ FEVE pré-operatório (%), angina instável prévia, balão intra-aórtico prévio, duração do BCP (minutos), e duração da anestesia (minutos). Parâmetros hemodinâmicos e metabólicos foram obtidos em cinco momentos: após a indução anestésica (INICIAL), no final da cirurgia (FINAL), na admissão na Unidade de Terapia Intensiva (UTI-1), seis horas após a admissão na UTI (UTI-6), e 12 horas após a admissão na UTI (UTI-12). O débito cardíaco foi adquirido pela técnica de hemodiluição, e amostras de sangue misto e arterial foram colhidas simultaneamente e analisadas (ABL 750; Radiometer, Copenhagen, Dinamarca) para a determinação das seguintes variáveis: tensão arterial de oxigênio (PaO_2_), tensão arterial de dióxido de carbono (PaCO_2_), tensão de oxigênio no sangue misto (PvO_2_), tensão de dióxido de carbono no sangue misto (PvCO_2_), saturação arterial de oxigênio (SaO_2_) e saturação de oxigênio no sangue venoso misto (SvO_2_). A concentração de hemoglobina (Hb) também foi medida. A concentração de oxigênio arterial (O_2_a), a concentração de dióxido de carbono (CO_2_) no sangue venoso misto (CO_2_v), a diferença arteriovenosa de oxigênio (_a-v_O_2_), a oferta de oxigênio (OO_2_), o consumo de oxigênio (VO_2_) e a taxa de extração de oxigênio (TEO_2_) foram calculadas utilizando-se fórmulas. A diferença venoarterial de CO_2_ (ΔCO_2_) e o Quociente Respiratório Estimado (QRe) foram calculados pela fórmula: ΔPCO_2_= PvCO_2_–PaCO_2_ eRQ= ΔPCO_2_/ C_a-v_O_2_.

Após a cirurgia, os pacientes foram admitidos na UTI pós-cirúrgica. Os desfechos em termos de tempo de ventilação mecânica, tempo de permanência hospitalar e na UTI, desfechos clínicos na UTI, e mortalidade hospitalar foram registrados.

Os pacientes foram divididos em dois grupos de acordo com a evolução clínica: grupo com desfecho negativo (óbito dentro de 30 dias após a cirurgia ou tempo de permanência na UTI superior a quatro dias) e grupo com desfecho positivo (tempo de permanência na UTI igual ou inferior a quatro dias) e alta hospitalar.

Complicações no pós-operatório foram definidas como: tempo de ventilação mecânica maior que 48 horas ou reintubação por qualquer motivo, complicações neurológicas caracterizadas por disfunção cognitiva aguda, isquemia cerebral documentada por tomografia computadorizada 48 horas após cirurgia cardíaca, insuficiência renal aguda definida por um aumento de 50% no valor anterior de creatinina sérica ou terapia renal substitutiva, e infecção, tais como pneumonia, mediastinite, infecção relacionada ao cateter ou bacteremia. Débito cardíaco baixo foi definido como necessidade de suporte inotrópico por mais de 24 horas ou um índice cardíaco menor que 2,2L/min/m^2^; embora um tratamento inotrópico tenha sido utilizado, não houve arritmias que necessitassem de intervenção farmacológica ou cardioversão elétrica.

### Análise estatística

A normalidade dos dados foi avaliada pelo teste de Shapiro-Wilk. As variáveis contínuas com distribuição normal foram descritas como média ± desvio padrão (DP) e aqueles sem distribuição normal foram descritas usando medianas e intervalos interquartis (IIQs).

O teste t não pareado ou o teste de Mann-Whitney foi usado para os dados contínuos conforme apropriado. As variáveis categóricas foram apresentadas como proporções e comparadas pelo teste do qui-quadrado ou o teste exato de Fisher. Uma análise de variância para medidas repetidas, seguida de um teste post-hoc de Bonferroni, foi usada para variáveis contínuas repetidas (variáveis hemodinâmicas e metabólicas) para determinar diferenças entre os dois grupos ao longo do estudo. Um modelo de regressão logística multivariada foi realizado pelo método *backwards* para identificar os fatores de risco independentes para permanência prolongada na UTI. Resultados de regressão logística são apresentados como razão de chance (odds ratio – OR) ajustada e intervalos de confiança de 95%. Curvas Características de Operação do Receptor (curvas ROC) foram construídas para identificar os pontos de corte ótimos associados com o desfecho. O ponto de corte ótimo foi definido como o valor associado com a soma mais alta da especificidade e sensibilidade. A área sob a curva ROC foi determinada e comparada. O programa GraphPrism (versão 5.0 GraphPad software, San Diego, CA, EUA) e o programa SPSS versão 15.0 (SPSS Inc., Chicago, Illinois, EUA) foram usados para as análises, e o nível de significância aplicado foi p<0,05.

## Resultados

### Dados clínicos e demográficos

Um total de 87 pacientes com idade entre 35 e 83 anos foram incluídos no estudo. Trinta e oito (43,7%) pacientes apresentaram desfechos adversos, e 49 pacientes (56,3%) apresentaram evolução normal ( Tabela 1 ). Pacientes com desfechos negativos eram mais velhos e apresentaram menores peso corporal e ASC em comparação aos pacientes com desfechos positivos. Pacientes com desfechos negativos apresentaram valores mais altos de EUROSCORE (mediana 6, IIQ 5-8) que aqueles com desfechos positivos (mediana 3, IIQ 3-5). As outras variáveis pré-operatórias e dados cirúrgicos não foram diferentes entre os grupos. O grupo que apresentou desfechos adversos apresentou um número significativo de complicações no pós-operatório ( Tabela 1 ).

**Tabela 1:** Dados demográficos, pré-operatórios, cirúrgicos, e pós-operatórios dos pacientes com evolução clínica complicada ou não complicada

Parâmetros	Evolução complicada (n= 49)	Evolução não complicada (n= 38)	p
**Demográficos**			
Idade (anos)	59,7 ± 9,4	66,3 ± 10	0,002 *
Peso (Kg)	74 (65,5-80)	65 (60-73,5)	0,005 *
ASC (m^2^)	1,81 ± 0,17	1,71 ± 0,16	0,006 *
Sexo feminino	12 (24,5%)	15 (39,5%)	0,13
**Pré-operatório**			
EuroSCORE	3 (3-5)	6 (5-8)	<0,001 *
FEVE (%)	40 (35-45,3)	42,5 (34,9-48)	0,40
BIA	3 (6,1%)	4 (10,5%)	0,71
**Cirúrgicos**			
Duração da cirurgia (min)	280 (240-340)	312 (245-374)	0,24
Duração da anestesia (min)	390 (320-430)	400 (334-480)	0,38
Tempo de BCP (min)	95 (75-118)	90 (69-140)	0,77
**Pós-operatório**			
Permanência na UTI (dias)	3 (2-4)	9 (6-20)	<0,001 *
Permanência no hospital (dias)	9 (7-13)	23 (14-29,5)	<0,001 *
Complicações respiratórias	21 (42,8%)	29 (76,3%)	0,002 *
Complicações neurológicas	0 (0%)	8 (21%)	0,002 *
Complicações renais	1 (2%)	18 (47,4%)	<0,001 *
Infecção	15 (30,6%)	28 (73,7%)	<0,001 *
Síndrome de baixo débito cardíaco	8 (16,3%)	34 (89,5%)	<0,001 *
Arritmias	8 (16,3%)	25 (65,8%)	<0,001 *

* Diferença estatisticamente significativa; dados apresentados como média ± desvio padrão, medianas (intervalo interquartil) ou valores absolutos (%); ASC: área de superfície corporal; FEVE: fração de ejeção do ventrículo esquerdo; BIA: uso prévio de Balão Intra-aórtico; BCP: Bypass Cardiopulmonar.

Variáveis hemodinâmicas e metabólicas medidas nos cinco momentos pré-estabelecidos foram comparadas entre ambos os grupos até a sexta hora de coleta (lactato) e a décima segunda hora de coleta de amostra (gradiente de CO_2_) ( Tabela 2 ).

**Tabela 2:** Variáveis metabólicas e hemodinâmicas dos pacientes de acordo com a evolução clínica no pós-operatório (média ± desvio padrão)

Parâmetro	Desfecho	Time				
		INICIAL	FINAL	UTI-1	UTI-6	UTI-12
QRe	Não complicado Complicado	1,35±0,58 1,36±0,65	1,82±0,68 1,91±0,90	1,69±0,69 1,70±0,97	1,51±0,84 1,59±0,61	1,41±0,67 1,86±0,82 *
ΔCO_2_ (mmHg)	Não complicado Complicado	6,11±2,98 6,01±2,62	5,81±1,81 5,51±2,40	6,20±2,26 6,45±3,60	6,30±3,11 6,76±2,78	5,55±2,86 7,75±3,10 **
Lactato (mmol/L)	Não complicado Complicado	1,57±0,58 1,67±0,73	3,77±1,82 4,96±3,21	3,86±2,19 5,17±3,54	2,63±1,45 4,26±2,63 **	2,04±0,83 3,05±2,31
BE	Não complicado Complicado	-0,49±1,90 -0,69±2,99	-4,45±2,49 -5,17±3,00	-4,74±3,22 -5,40±3,47	-4,09±2,97 -5,20±4,15	-3,44±2,43 -3,82±3,61
Svo_2_ (%)	Não complicado Complicado	74,58±7,50 73,32±8,87	77,29±4,59 77,87±6,77	73,68±6,41 72,05±8,44	65,92±7,09 66,19±8,21	68,44±6,69 67,19±7,51
_a-v_O_2_ (mL/dL)	Não complicado Complicado	4,56±1,26 4,72±1,75	3,30±0,69 3,01±0,75	3,80±0,95 3,92±0,99	4,34±0,89 4,44±1,17	4,01±1,08 4,38±1,09
OO_2_ (mL/min)	Não complicado Complicado	485,0±212,0 467,2±186,5	813,0±297,8 727,7±163,5	566,0±241,7 625,3±294,5	788,8±252,8 763,5±282,3	869,4±291,3 744,3±197,9
VO_2_ (mL/min)	Não complicado Complicado	125,7±59,25 131,3±67,88	194,8±73,13 164,1±40,31	146,9±64,98 161,7±84,34	243,4±74,63 234,2±73,49	253,8±80,55 228,0±47,24
TEO_2_ (%)	Não complicado Complicado	26,44±7,30 28,07±8,06	24,69±5,92 23,18±5,76	26,05±6,37 27,94±7,96	31,80±6,56 32,08±8,17	30,37±6,74 32,07±7,09
IC (L/min/m^2^)	Não complicado Complicado	2,40±0,68 2,34±0,73	3,52±1,04 3,31±0,61	3,06±0,78 3,03±0,88	3,21±0,77 3,16±1,01	3,62±0,91 3,17±0,71

* p<0,05 entre grupos;

** p<0.01 between groups.

INITIAL: após indução anestésica; FINAL: final da cirurgia, UTI-1: admissão na Unidade de Terapia Intensiva (UTI); UTI-6: seis horas após a admissão na UTI; UTI-12: 12 horas após a admissão na UTI (UTI-12); QRe: quociente respiratório estimado; ΔPCO2: gradiente venoarterial de dióxido de carbono; SvO2: saturação venosa central de oxigênio; a-vO2: diferença arteriovenosa de oxigênio; OO2: oferta de oxigênio; VO2: consumo de oxigênio (VO2); TEO2: taxa de extração de oxigênio; IC: índice cardíaco.

### Quociente respiratório estimado e ΔpCO_2_

Pacientes com complicações clínicas apresentaram valores de QRe significativamente mais altos 12 horas após a admissão na UTI. Resultados similares foram observados para ΔCO_2_, que também foram mais altos no tempo UTI-12 naquele grupo. ( Tabela 2 ).

### Lactato

Valores de lactato arterial não foram diferentes entre os grupos em nenhum dos tempos avaliados exceto às seis horas após a admissão na UTI (UTI-6), quando pacientes com piores desfechos apresentaram níveis significativamente mais altos desse biomarcador ( Tabela 2 ).

*Excesso de base, Sv* *O*_2_, _a-v_ *O*_2_ *, O* *O*_2_ *, V* *O*_2_, TEO_2_ *e índice cardíaco* Esses parâmetros não foram diferentes entre os grupos em nenhum dos tempos avaliados ( Tabela 2 ).

### Modelo multivariado para identificar determinantes independentes de permanência prolongada na UTI

As variáveis identificadas como significativamente diferentes entre os grupos (EuroSCORE, peso corporal, UTI-12 QRe, UTI-12 ΔPCO_2_ e lactate em UTI-6) foram incluídos neste modelo para identificar determinantes independentes de permanência prolongada na UTI. As idades dos pacientes foram distribuídas em cinco grupos para análises: ≤ 50 anos, 51 a 60 anos, 61 a 70 anos, 71 a 80 anos, e ≥ 81 anos.

No primeiro modelo de análise, "ΔPCO_2_ em UTI012" foi excluída porque existe uma relação matemática entre ΔPCO_2_ e QRe que poderia resultar em colinearidade e uma modificação dos resultados. Uma análise de regressão logística por *stepwise* mostrou que Euro SCORE, e medidas de lactato nos tempos UTI-12 e UTI-6 foram independentemente associados com uma evolução complicada no pós-operatório ( Tabela 3 ).

**Tabela 3:** Regressão logística na predição de uma evolução clínica complicada após a cirurgia de bypass coronário em pacientes com disfunção ventricular esquerda –primeiro modelo: sem o gradiente venoarterial de dióxido de carbono (ΔCO2), medido 12 horas após a admissão da unidade de terapia intensiva

Variável	Regressão logística univariada			Regressão logística multivariada		
	Odds ratio	IC95%	p univariado	Odds ratio	IC95%	p multivariado
Grupo etário (10 anos)	1,77	0,67 a 4,69	0,249	-	-	-
Peso (kg)	0,95	0,88 a 1,01	0,124	-	-	-
EuroSCORE	2,25	1,35 a 3,77	0,002	2,50	1,58 a 3,97	<0,001
Lactato UTI-6 (mmol/l)	1,68	1,10 a 2,56	0,017	1,81	1,16 a 2,83	0,009
QRe UTI-12 (mmHg/mL/dL)	4,11	1,26 a 13,34	0,019	3,21	1,16 a 8,86	0,024

Grupo etário: cada 10 anos acima dos 50 anos de idade; UTI-6: seis horas após a admissão na unidade de terapia intensiva; QRe: quociente respiratório estimado; UTI-12: 12 horas após a admissão na unidade de terapia intensiva; IC: intervalo de confiança.

No segundo modelo de análise, "ΔPCO_2_ no tempo UTI-12" foi incluído, e o "QRe no UTI-12" excluído. Resultados similares foram observados para Euro SCORE, lactato em UTI-6 e ΔPCO_2_ em UTI-12, que foram independentemente associados com uma evolução complicada ( Tabela 4 ).

**Tabela 4:** Regressão logística na predição de uma evolução clínica complicada após a cirurgia de *bypass* coronário em pacientes com disfunção ventricular esquerda (segundo modelo – sem quociente respiratório em T4

Variáveis	Regressão logística univariada			Regressão logística multivariada		
	Odds ratio	IC95%	p univariado	Odds ratio	IC95%	p multivariado
Grupo etário (10 anos)	2,13	0,75 a 6,05	0,154	-	-	-
Peso (kg)	0,94	0,88 a 1,01	0,114	-	-	-
EuroSCORE	2,19	1,32 a 3,63	0,002	2,51	1,58 a 3,97	<0,001
Lactato UTI-6 (mmol/l)	1,61	1,07 a 2,43	0,022	1,68	1,10 a 2,56	0,016
ΔCO_2_ UTI-12 (mmHg)	1,47	1,10 a 1,95	0,009	1,33	1,05 a 1,68	0,015

Grupo etário: cada 10 anos acima dos 50 anos de idade; UTI-6: seis horas após a admissão na unidade de terapia intensiva; QRe: quociente respiratório estimado; UTI-12: 12 horas após a admissão na unidade de terapia intensiva; ΔCO_2_: gradiente venoarterial de dióxido de carbono; IC: intervalo de confiança.

### Curvas ROC

Os valores ótimos para os pontos de corte de Euro SCORE, "ΔPCO_2_ no tempo UTI-12"_,_ "QRe no UTI-12," e "lactato no UTI-6" para identificar pacientes com desfechos complicados no pós-operatório foram definidos pela análise das curvas ROC ( Tabela 5 ). As áreas sub as curvas ROC das variáveis não foram significativamente diferentes.

**Tabela 5:** Áreas sob Curvas Características de Operação do Receptor (curvas ROC) para predizer uma evolução clínica complicada após a cirurgia de bypass coronário em pacientes com disfunção ventricular esquerda

Parâmetro	Área	IC95%	p	Ponto de corte	Sensibilidade (%)	Especificidade (%)
EURO SCORE	0,76	0,66 a 0,86	<0,0001	5	78	65
Lactato UTI-6 (mmol/l)	0,67	0,55 a 0,80	0,0033	4	49	84
QRe UTI-12 (mmHg/mL/dL)	0,68	0,57 a 0,80	0,0010	1,65	54	78
ΔCO_2_ UTI-12 (mmHg)	0,72	0,60 a 0,83	<0,0001	6,9	62	80

UTI-6: seis horas após a admissão na unidade de terapia intensiva; QRe: quociente respiratório estimado; UTI-12: 12 horas após a admissão na unidade de terapia intensiva; ΔCO_2_: gradiente venoarterial de dióxido de carbono; IC: intervalo de confiança.

Um EuroSCORE ≥5 foi preditor de uma pior evolução com uma sensibilidade de 78% e uma especificidade de 65%. Um QRe ≥ 1,65 mmHg/mL/dL 12 horas após a admissão na UTI foi preditor de um desfecho complicado, com uma sensibilidade de 54% e uma especificidade de 78%. Uma ΔPCO_2_ ≥ 69 mmHg no tempo UTI-12 foi preditor de uma evolução complicada, com uma sensibilidade de 62% e uma especificidade de 80%, e lactato no tempo UTI-6 ≥ 4mmol/L foi preditor de complicações, com uma sensibilidade de 49% e uma especificidade de 84% ( Tabela 5 ).

## Discussão

O objetivo do presente estudo foi avaliar se marcadores perioperatórios de hipoperfusão tecidual são preditores de desfechos adversos no período pós-operatório em pacientes com disfunção ventricular esquerda submetidos à cirurgia de *bypass* coronário. Os principais resultados indicaram que o EuroSCORE, o lactato arterial seis horas após o procedimento QRe e ΔpCO_2_ são preditores independentes de uma permanência prolongada na UTI.

Pacientes com disfunção ventricular esquerda submetidos à cirurgia de *bypass* coronário geralmente apresentam taxas mais altas de complicações,^13^ como observado em nosso estudo, resultando em uma permanência prolongada na UTI. A predição precoce de uma evolução complicada no pós-operatório pode requerer atenção especial para minimizar complicações.

Em nossos dados, foi possível identificar que alguns biomarcadores da perfusão tecidual foram diferentes na primeira hora e às 12 horas no grupo de pacientes que apresentaram uma evolução favorável. Nossos dados sugerem que um QRe 12 horas após a cirurgia é um marcador confiável de distúrbios de oxigênio no tecido relacionados a complicações. O QR tem sido estudado em diferentes situações clínicas para indicar hipóxia tecidual e metabolismo anaeróbico.^5 , 7 , 16 – 18^ Contudo, seu papel como um marcador de desfecho ainda não está bem definido. Mekontso-Dessap et al.^5^ estudaram pacientes críticos e sugeriram que o QRe maior que 1,4 mmHg/mL/dL estima uma menor sobrevida global em um mês.^5^ Lundin et al.^16^ mostraram que, 24 horas após uma parada cardíaca, os níveis de QR forma independentemente associados com mortalidade na UTI mas não com o desfecho neurológico três meses depois. Por outro lado, ΔpCO2 associou-se com uma taxa mais baixa de mortalidade na UTI e um pior desfecho neurológico. Entre ScvO2 e ΔpCO2, somente ΔpCO2 foi capaz de predizer um pior desfecho.^16^

O presente estudo avaliou o valor preditivo do QRe em pacientes de cirurgia cardíaca de alto risco. Nossos resultados sugerem que um QRe maior que 1,65 mmHg/mL/dL poderia predizer complicações no pós-operatório, com uma sensibilidade de 54% e uma especificidade de 78% quando analisado 12 horas após admissão na UTI.

Um QR alto está relacionado com metabolismo anaeróbico quando a OO_2_ é inadequada para atender a demanda de O2 (VO_2_). A resposta fisiológica à OO_2_ diminui nos tecidos e leva a um amento na TEO_2_ do sangue capilar para manter a produção de adenosina trifostato (ATP) e a demanda de energia celular. Contudo, com reduções críticas na OO_2_, aumentos compensatórios na TEO_2_ pode não ser suficiente para fornecer o oxigênio necessário para manter o metabolismo aeróbico. Nesse contexto, reduções no VO_2_ celular causam a produção anaeróbica de CO_2_. Essa complexa equação entre o fornecimento e a demanda de oxigênio explica o aumento no (VCO_2_/VO_2_) quando a OO_2_ cai para níveis críticos.^17^

A ΔpCO_2_ elevada analisada 12 horas após a admissão na UTI e a alta concentração ode lactato arterial seis horas após a cirurgia também se associaram com piores desfechos. Os valores de outros marcadores de perfusão – BE, SvO_2, a-v_O_2_, OO_2_, VO_2_, TEO_2_ e índice cardíaco não foram diferentes.

O mecanismo fisiológico da elevação na diferença venoarterial de CO_2_ (ΔPCO_2_) para estar mais relacionado com situações de baixo fluxo sanguíneo do que com hipóxia tecidual e produção anaeróbica de CO_2_. Vallet et al.^19^ demonstraram, em um modelo experimental de hipóxia isquêmica e hipóxica que a diminuição da OO_2_ pela redução do fluxo sanguíneo resultou em um aumento no ΔPCO_2_, ao passo que diminuindo-se a OO_2_ pela redução da oxigenação sanguínea não afetou o ΔPCO_2_.^19^ O fenômeno de estagnação de CO_2_ pode explicar a ampliação da ΔPCO_2_ em situações de baixo fluxo sanguíneo. O tempo de trânsito do fluxo sanguíneo ficou mais lento, resultando em um maior incremento de CO_2_ por unidade de sangue percorrendo os microvasos eferentes e gerando hipercarbia venosa.^20^

Um alto valor da diferença venoarterial de pCO_2_ tem sido relacionado a um desfecho desfavorável em diferentes cenários clínicos.^9 , 21^ Na cirurgia cardíaca, Cavaliere et al.^9^ observaram uma associação entre ΔPCO_2_ elevada e complicações pós-operatórias. Dificuldade no desmame do BCP também foi relacionada a valores elvados de ΔPCO_2,_ como demonstrado por Denault et al.^22^ De acordo com nossos resultados, um ΔPCO_2_ maior que 6,9 mmHg correlaciona-se com um desfecho pós-operatório negativo. Curvas ROC apresentaram 62% de sensibilidade e 80% de especificidade na análise da ΔPCO_2_ 12 horas após a admissão na UTI.

Um dado interessante é que valores iniciais de ΔPCO_2_ QRe na admissão na UTI e seis horas depois não foram associados com desfechos em nossos pacientes. Esses achados sugerem que os valores iniciais dos parâmetros de perfusão tecidual não tiveram a mesma capacidade preditiva dos valores posteriores quando associados à evolução clínica. A inflamação nesses pacientes, que foi mais pronunciada nas primeiras horas após o BCP, resultou em maior fluxo sanguíneo e poderia explicar por que o QRe apresentou maior relação com a ΔPCO_2_ que o lactato arterial. Essa elevação no QRe pode ser explicada pela equação matemática incluindo ΔPCO_2_ do oque com hipóxia tecidual e metabolismo anaeróbico.

Níveis elevados de lactato plasmático também foram correlacionados com uma permanência prolongada na UTI em nosso estudo. A hiperlactatemia é um marcador bem reconhecido de insuficiência circulatória, e seus valors foram associados com aumento na mortalidade em várias situações clínicas.^3 , 8 , 23 , 24^ Durante a cirurgia cardíaca com BCP, uma alta concentração de lactato está frequentemente (10 a 20%) associada à morbidade e à mortalidade pós-operatória.^3 , 25^ As causas de hiperlactatemia durante e após a cirurgia cardíaca permanecem controversas. A maioria dos autores atribuem esse achado à hipóxia tecidual (hiperlactatemia tipo A). No entanto, a hiperlactatemia tipo B, que ocorrem em pacientes sem hipóxia tecidual, pode ocasionalmente ser vista após o BCP.^3 , 26 , 27^ Neste estudo, a concentração de lactato arterial aumentou após a cirurgia e permaneceu elevada até a admissão na UTI, quando diminuiu progressivamente. Os níveis de lactato seis horas após a cirurgia foram significativamente mais altos no grupo com tempo na UTI prolongado. Essa diferença não foi mantida, sugerindo que a elevação no lactato se relacionou à hipoperfusão tecidual intraoperatória, que contribuiu para um curso desfavorável na nossa população. De acordo com nossos resultados, uma concentração de lactato arterial ≥ 4 mmol/L no tempo UTI-6 foi preditor de um desfecho complicado com uma sensibilidade de 49% e uma especificidade de 84%. Essa ausência de sensibilidade foi identificada em um estudo prévio^28^ e sugere que níveis iniciais de lactato não podem predizer certos eventos adversos pós-operatórios.

Pacientes com desfechos desfavoráveis também apresentaram um EuroSCORE mais alto. A correlação entre o EuroSCORE e a evolução complicada na UTI foi desctit,^15 , 29^ o que enfatiza a importância do EuroSCORE como um parâmetro de rastreamento para predizer o tempo de permanência da UTI.

Como demonstrado em estudos anteriores,^30 , 31^ parâmetros derivados de oxigênio apresentam baixa correlação com metabolismo anaeróbico e, portanto, não podem ser usados como indicadores prognósticos em nossa população específica. A interpretação para um baixo VO_2_ é difícil; ele pode estar relacionado à hipóxia tecidual ou a uma demanda reduzida de O2 sem hipóxia sistêmica ou a uma baixa temperatura. Valores baixos de SvO_2_ podem estar associados com hipóxia tecidual global, com uma diminuição aguda na OO_2_, ou com condições aeróbicas se mecanismos compensatórios da extração de O_2_ forem inadequadas. Por outro lado, valores médios ou mesmo altos de SvO_2_ podem estar associados à hipóxia tecidual profunda relacionada a uma deficiente extração de oxigênio ou baixo metabolismo causado por anestesia profunda e hipotermia. Um desequilíbrio de temperatura em diferentes compartimentos do corpo devido a um reaquecimento não uniforme após o bypass^32^ pode às vezes explicar nos valores iniciais altos de SvO_2_. Conclusões similares podem ser tiradas para O_2_ER e _a-v_O_2_. De fato, não encontramos diferenças significativas entre SvO_2_, DO_2_, VO_2_, O_2_ER, e C_a-v_O_2_.

### Limitações do estudo

As limitações de nosso estudo devem ser mencionadas. Os fatores de confusão podem haver influenciado os resultados obtidos; por exemplo, o estado inflamatório que causa elevação anormal no débito cardíaco e otimização hemodinâmica inadequada. Níveis elevados de QRe, ΔPCO_2,_ lactato, e EuroSCORE no pós-operatório foram significativamente correlacionados com uma evolução complicada após a cirurgia. Contudo, não podemos concluir se essa elevação nesses parâmetros de hipoperfusão foi relacionada com uma otimização insuficiente com drogas vasoativas ou à refratariedade ao tratamento. Assim, a otimização da perfusão tecidual em pacientes de alto risco submetidos à cirurgia cardíaca deveria ser estudada usando valores ótimos de QRe, ΔPCO_2,_ e lactato como alvos.

## Conclusão

Nossos achados mostraram que EuroSCORE, lactato arterial seis horas após a cirurgia, ΔPCO2 12 horas após a cirurgia, e QRe são preditores independentes de eventos adversos em pacientes com disfunção ventricular esquerda após a cirurgia cardíaca. O poder preditivo desses parâmetros de hipoperfusão independe de fatores pré-operatórios representados pelo EuroSCORE. Não houve superioridade de nenhum biomarcador identificado como um preditor independente.
