# Supplementary material for: Marcadores de Perfusão Tecidual como Preditores de Desfechos Adversos em Pacientes com Disfunção Ventricular Esquerda Submetidos à Revascularização Miocárdica (Bypass Coronário)
Source: Arq Bras Cardiol. 2024 Mar 13;121(2):e20230247. [Article in Portuguese] doi: 10.36660/abc.20230247 (PMC12092063; doi:10.36660/abc.20230247)
Supplement: Translation

# Translation: Markers of Tissue Perfusion as Predictors of Adverse Outcomes in Patients with Left Ventricular Dysfunction Undergoing Coronary Artery Bypass Surgery

**Authors:** Thiana Yamaguti, José Otavio Costa Auler, Luís Alberto Oliveira Dallan, Filomena Regina Barbosa Gomes Galas, Ligia Cristina Câmara Cunha, Marilde de Albuquerque Piccioni

**Affiliations:** 1 São Paulo SP Brazil Instituto do Coração do Hospital das Clínicas da Faculdade de Medicina da Universidade de São Paulo, São Paulo, SP – Brazil

**Keywords:** Thoracic Surgery, Left Ventricular Dysfunction, Biomarkers

## Abstract

**Background:**

Cardiac surgery patients may be exposed to tissue hypoperfusion and anaerobic metabolism.

**Objective:**

To verify whether the biomarkers of tissue hypoperfusion have predictive value for prolonged intensive care unit (ICU) stay in patients with left ventricular dysfunction who underwent coronary artery bypass surgery.

**Methods:**

After approval by the institution's Ethics Committee and the signing of informed consent, 87 patients with left ventricular dysfunction (ejection fraction < 50%) undergoing coronary artery bypass surgery were enrolled. Hemodynamic and metabolic biomarkers were collected at five time points: after anesthesia, at the end of the surgery, at ICU admission, and at six and twelve hours after. An analysis of variance for repeated measures followed by a Bonferroni post hoc test was used for repeated, continuous variables (hemodynamic and metabolic variables) to determine differences between the two groups over the course of the study period. The level of statistical significance adopted was 5%.

**Results:**

Thirty-eight patients (43.7%) who presented adverse outcomes were older, higher Euro score (p<0.001), and elevated ΔpCO_2_as analyzed 12 hours after ICU admission (p<0.01), while increased arterial lactate concentration at 6 hours postoperatively was found to be a negative predictive factor (p<0.01).

**Conclusions:**

Euro SCORE, six-hour postoperative arterial lactate, 12-hour postoperative ΔPCO_2_, and eRQ are independent predictors of adverse outcomes in patients with left ventricular dysfunction after cardiac surgery.

## Introduction

Patients who undergo cardiac surgery may be exposed to tissue hypoperfusion and anaerobic metabolism.^1^The factors involved in cellular hypoxemia are hemodilution, low cardiac output, and inflammatory reactions following cardiopulmonary bypass (CPB).^2 – 4^The biomarkers of tissue hypoperfusion, such as lactate levels, venoarterial CO_2_gradient (ΔPCO_2_), estimated respiratory quotient (eRQ), and central venous oxygen saturation (SvO_2_), are increased by low cardiac output.^3 , 5 – 8^Lactate levels during shock states are increased due to tissue hypoxia, increased glucose metabolism, and decreased lactate clearance.^1^The ΔPCO_2_increases with low cardiac output or inadequate microcirculatory perfusion that means blood flow stagnation in the venous compartment.^6^Furthermore, eRQ (ΔPCO_2_corrected by the arterial-to-venous oxygen content difference) has demonstrated better sensibility for anaerobic metabolism detection when compared with ΔPCO_2_alone.^7^

Nevertheless, tissue hypoperfusion could be masked by the inflammatory response and vasodilatory states following bypass.^8 – 11^Patients with left ventricular dysfunction under cardiac surgery are more likely to present low cardiac output syndrome associated with a systemic inflammatory response.^12^This situation results in a prolonged stay in the intensive care unit (ICU) and higher mortality rates as compared to patients with normal ventricular function.^13^The early detection of tissue hypoperfusion may predict adverse outcomes in this group of patients who contribute to prolonged ICU stay and high mortality. Optimizing the hemodynamic profile based on certain values of cardiac index and oxygen delivery to the cells is the main rule in postoperative management. The association of cardiac output measurement and biomarkers of tissue perfusion may define the support with vasoactive drugs and volume adjustment. The question addressed in this study is whether the biomarkers of tissue hypoperfusion have a predictive value for prolonged ICU stay in patients with left ventricular dysfunction who underwent coronary artery bypass surgery.

## Methods

This is a prospective observational study conducted over two years at the InCor- Heart Institute of the General Hospital of the University of Sao Paulo Medical School. All the patients gave written consent, and the Local Ethical Committee approved the study (CAPPESQ n. 0517/04).

### Patients

This was a prospective observational cohort study conducted during 24 consecutive months. During this period, 183 patients with left ventricular dysfunction (ejection fraction < 50%) were submitted to coronary bypass graft surgery and 110 patients were eligible in the study. Twenty-three patients were excluded. Left ventricular ejection fraction (LVEF) was measured preoperatively using 2D echocardiography. The exclusion criteria were renal failure (creatinine clearance lower than 40 mL/min/m^2^), hepatic dysfunction, endocrine disorders, pulmonary disease, uncontrolled diabetes mellitus, a history of fever or infection within the week before surgery, and previous anemia (hemoglobin ≤ 10.0 g/dL).

### Anesthesia, surgery, and CPB

Anesthesia was induced with 3-5 μg/kg fentanyl, 0.05 mg/kg midazolam, etomidate 0.2-0.3 mg/kg, muscle relaxation, and fentanyl and sevoflurane for maintenance. Standard monitoring for cardiac surgery was used, and a pulmonary artery catheter was introduced for hemodynamic monitoring. An initial 500 IU kg dose of heparin was administered for anticoagulation, and the activated clotting time was fixed at 480 seconds. At the end of CPB, heparin was reversed by protamine chloride at a 1:1 ratio of the loading dose, regardless of the total heparin dosage. Blood flow during CPB was set between 2.0 and 2.4 L/m^2^or according to the mean arterial pressure held at approximately 60 mmHg. A membrane oxygenator was used in all patients, and intermittent cold blood cardioplegia (every 10 minutes, anterograde) was used for myocardial protection. During CPB, the core temperature was maintained at 32-34 °C, and hematocrit levels were between 22% and 25%.

### Data collection and definitions

The demographic, clinical and operative data recorded were age (years), sex, weight (Kg), body surface area (BSA, m^2^), Euro SCORE,^14 , 15^preoperative LVEF (%), previous unstable angina, previous intra-aortic balloon (IAB), CPB duration (minutes), surgery and anesthesia duration (minutes). Hemodynamic and metabolic parameters were obtained at five time points: after anesthesia induction (INITIAL), at the end of surgery (FINAL), at admission to the postsurgical intensive care unit (ICU) (ICU-1), six hours after ICU admission (ICU-6), and 12 hours after ICU admission (ICU-12). Cardiac output was acquired by the thermodilution technique, and arterial and mixed venous blood samples were withdrawn simultaneously and analyzed (ABL 750; Radiometer, Copenhagen, Denmark) for determination of the following variables: arterial oxygen tension (PaO_2_), arterial carbon dioxide tension (PaCO_2_), mixed venous oxygen tension (PvO_2_), mixed venous carbon dioxide (CO_2_) tension (PvCO_2_), arterial oxygen saturation (SaO_2_) and mixed venous oxygen saturation (SvO_2_). Hemoglobin concentration (Hb) was also measured. The arterial oxygen content (CaO_2_), mixed venous oxygen content (CvO_2_), arteriovenous oxygen content difference (C_a-v_O_2_), oxygen delivery (DO_2_), oxygen consumption (VO_2_), and oxygen extraction ratio (O_2_ER) were calculated using standard formulas. The veno-arterial CO_2_tension difference (ΔPCO_2_) and estimated respiratory quotient (eRQ) were calculated with the following formula: ΔPCO_2_= PvCO_2_–PaCO_2_eRQ= ΔPCO_2_/ C_a-v_O_2_.

After surgery, patients were admitted to the postsurgical ICU. The outcomes in terms of mechanical ventilation time, ICU and hospital length of stay, clinical outcomes in the ICU, and hospital mortality were recorded.

Patients were divided into two groups according to their postoperative clinical course: the negative outcome group (death within 30 days after surgery or ICU stay > 4 days) and the positive outcome group (ICU stay ≤ 4 days) and hospital discharge.

Postoperative complications were defined as follows: mechanical ventilation longer than 48 hours or reintubation for any reason, neurologic complications characterized by acute cognitive dysfunction, brain ischemia as documented by computed tomography scan 48 hours after cardiac surgery, acute renal failure defined as an increase of 50% in the previous value of serum creatinine or renal replacement therapy, and infection, such as pneumonia, mediastinitis, catheter-related infection, or bacteremia. Low cardiac output was defined as requiring inotropic support for more than 24 hours or a cardiac index lower than 2.2 L/min/m^2^; although inotropic treatment was used, there were no arrhythmias that required pharmacological intervention or electric cardioversion.

### Statistical analysis

Data normality was assessed by Shapiro-Wilk test. Continuous variables with normal distribution were described through mean ± standard deviation (SD) and those without normal distribution were described using median and interquartile range.

Unpaired t test or Mann–Whitney *U* test was used for continuous data as appropriate. Categorical variables were presented as proportions and were compared with the chi-square test or Fisher's Exact test. An analysis of variance for repeated measures followed by a Bonferroni *post hoc* test was used for repeated, continuous variables (hemodynamic and metabolic variables) to determine differences between the two groups over the study period. A multivariate logistic regression model was performed backwards to identify the independent risk factors for prolonged ICU stay. Logistic regression results are reported as adjusted odds ratios (OR) with 95% confidence intervals. Receiver operator characteristic (ROC) curves were constructed to identify optimal cutoff values associated with the outcome. The optimum cutoff was defined as the value associated with the highest sum of sensitivity and specificity. The area under the ROC curve was determined and compared. GraphPad Prism version 5.0 for Windows (GraphPad Software, San Diego, CA, USA) and SPSS version 15.0 (SPSS Inc., Chicago, Illinois, USA) were used for analyses, and significance was set at p < 0.05.

## Results

### Demographic and clinical data

A total of 87 patients aged 35 to 83 years were included in this study. Thirty-eight patients (43.7%) presented with some adverse outcomes, and 49 patients (56.3%) presented with normal evolution ( Table 1 ). Patients with negative outcomes were older and presented lower body weight and BSA compared to those with positive outcomes. Patients with negative outcomes had higher values of EUROSCORE (median 6, IQR 5-8) than those with positive outcomes (median 3, IQR 3-5). The other preoperative variables and surgical data were not different between the groups. The group that had adverse outcomes had a significant number of postoperative complications ( Table 1 ).

**Table 1:** Demographic, preoperative, surgical, and postoperative data of patients with complicated clinical courses and uncomplicated courses

Parameters	Uncomplicated course (n= 49)	Complicated course (n= 38)	p
**Demographic**			
Age (years)	59.7 ± 9.4	66.3 ± 10	0.002 *
Weight (kg)	74 (65.5-80)	65 (60-73.5)	0.005 *
BSA (m^2^)	1.81 ± 0.17	1.71 ± 0.16	0.006 *
Female	12 (24.5%)	15 (39.5%)	0.13
**Preoperative**			
EuroSCORE	3 (3-5)	6 (5-8)	<0.001 *
LVEF (%)	40 (35-45.3)	42.5 (34.9-48)	0.40
IAB	3 (6.1%)	4 (10.5%)	0.71
**Surgical**			
Surgery duration (min)	280 (240-340)	312 (245-374)	0.24
Anesthesia duration (min)	390 (320-430)	400 (334-480)	0.38
CPB time (min)	95 (75-118)	90 (69-140)	0.77
**Postoperative**			
ICU length of stay (days)	3 (2-4)	9 (6-20)	<0.001 *
Hospital length of stay (days)	9 (7-13)	23 (14-29.5)	<0.001 *
Respiratory complications	21 (42.8%)	29 (76.3%)	0.002 *
Neurologic complications	0 (0%)	8 (21%)	0.002 *
Renal complications	1 (2%)	18 (47.4%)	<0.001 *
Infection	15 (30.6%)	28 (73.7%)	<0.001 *
Low cardiac output syndrome	8 (16.3%)	34 (89.5%)	<0.001 *
Arrhythmias	8 (16.3%)	25 (65.8%)	<0.001 *

* Statistically significant difference. Data are presented as the means ± standard deviation, medians (interquartile range) or absolute values (%); BSA: body surface area; LVEF: left ventricular ejection fraction; IAB: previous use of intraaortic balloon counterpulsation; CPB: cardiopulmonary bypass.

Hemodynamic and metabolic variables measured at the five preestablished time points were compared between both groups up to the sixth hour of collection (lactate) and the twelfth hour of sample collection (CO_2_gradient) ( Table 2 ).

**Table 2:** Hemodynamic and metabolic variables of patients according to the postoperative clinical course (mean ± SD)

Parameter	Outcome	Time				
		INITIAL	FINAL	ICU-1	ICU-6	ICU-12
eRQ	Uncomplicated Complicated	1.35±0.58 1.36±0.65	1.82±0.68 1.91±0.90	1.69±0.69 1.70±0.97	1.51±0.84 1.59±0.61	1.41±0.67 1.86±0.82 *
ΔCO_2_(mmHg)	Uncomplicated Complicated	6.11±2.98 6.01±2.62	5.81±1.81 5.51±2.40	6.20±2.26 6.45±3.60	6.30±3.11 6.76±2.78	5.55±2.86 7.75±3.10 **
Lactate (mmol/L)	Uncomplicated Complicated	1.57±0.58 1.67±0.73	3.77±1.82 4.96±3.21	3.86±2.19 5.17±3.54	2.63±1.45 4.26±2.63 **	2.04±0.83 3.05±2.31
BE	Uncomplicated Complicated	-0.49±1.90 -0.69±2.99	-4.45±2.49 -5.17±3.00	-4.74±3.22 -5.40±3.47	-4.09±2.97 -5.20±4.15	-3.44±2.43 -3.82±3.61
Svo_2_(%)	Uncomplicated Complicated	74.58±7.50 73.32±8.87	77.29±4.59 77.87±6.77	73.68±6.41 72.05±8.44	65.92±7.09 66.19±8.21	68.44±6.69 67.19±7.51
C_a-v_O_2_(mL/dL)	Uncomplicated Complicated	4.56±1.26 4.72±1.75	3.30±0.69 3.01±0.75	3.80±0.95 3.92±0.99	4.34±0.89 4.44±1.17	4.01±1.08 4.38±1.09
DO_2_(mL/min)	Uncomplicated Complicated	485.0±212.0 467.2±186.5	813.0±297.8 727.7±163.5	566.0±241.7 625.3±294.5	788.8±252.8 763.5±282.3	869.4±291.3 744.3±197.9
VO_2_(mL/min)	Uncomplicated Complicated	125.7±59.25 131.3±67.88	194.8±73.13 164.1±40.31	146.9±64.98 161.7±84.34	243.4±74.63 234.2±73.49	253.8±80.55 228.0±47.24
O_2_ER (%)	Uncomplicated Complicated	26.44±7.30 28.07±8.06	24.69±5.92 23.18±5.76	26.05±6,37 27.94±7.96	31.80±6.56 32.08±8.17	30.37±6.74 32.07±7.09
CI (L/min/m^2^)	Uncomplicated Complicated	2.40±0.68 2.34±0.73	3.52±1.04 3.31±0.61	3.06±0.78 3.03±0.88	3.21±0.77 3.16±1.01	3.62±0.91 3.17±0.71

* p<0.05 between groups;

** p<0.01 between groups.

INITIAL: after anesthesia induction, FINAL: end of surgery; ICU-1: admission to the postsurgical ICU, ICU-6: 6 hours after ICU admission, ICU-12: 12 hours after ICU admission.

### Estimated respiratory quotient and ΔpCO_2_

Patients with clinical complications presented significantly higher eRQ values 12 hours after ICU admission. Similar results were observed for ΔPCO_2_, which was also higher at ICU-12 in that group ( Table 2 ).

### Lactate

Values of arterial lactate were not different between groups at all time points except at six hours after ICU admission (ICU-6), when patients with worse outcomes had significantly higher levels of this biomarker ( Table 2 ).

*Base excess, SvO*_2_ *, C*_a-v_ *O*_2_ *, DO*_2_ *, VO*_2_ *, O*_2_ *ER and cardiac index* These parameters were not different between groups at any established moments ( Table 2 ).

### Multivariate model to identify independent determinants of prolonged ICU stay

Variables identified to be significantly different between groups (EuroSCORE, body weight, ICU-12 eRQ, ICU-12 ΔPCO_2_and ICU-6 lactate) were included in this model to identify independent determinants of prolonged ICU stay. Patient ages were distributed in five clusters for analysis: ≤ 50 years, 51 to 60 years, 61 to 70 years, 71 to 80 years, and ≥ 81 years.

In the first model of analysis, "ICU-12 ΔPCO_2_" was excluded because there is a mathematical relation between ΔPCO_2_and eRQ that could result in collinearity and a modification of the results. A stepwise logistic regression analysis showed that Euro SCORE, ICU-12 and ICU-6 lactate were all independently associated with complicated course in the postoperative period ( Table 3 ).

**Table 3:** Logistic regression for predicting a complicated postoperative clinical course after coronary artery bypass surgery in patients with left ventricular dysfunction; first model: without venoarterial CO_2_gradient (ΔCO_2_) at 12 hours after intensive care unit admission

Variable	Univariate logistic regression			Multivariate logistic regression		
	Odds ratio	95% CI	Univariate p	Odds ratio	95% CI	Multivariate p
Age group (10 years)	1.77	0.67 to 4.69	0.249	-	-	-
Weight (kg)	0.95	0.88 to 1.01	0.124	-	-	-
EuroSCORE	2.25	1.35 to 3.77	0.002	2.50	1.58 to 3.97	<0.001
Lactate ICU-6 (mmol/l)	1.68	1.10 to 2.56	0.017	1.81	1.16 to 2.83	0.009
eRQ ICU-12 (mmHg/mL/dL)	4.11	1.26 to 13.34	0.019	3.21	1.16 to 8.86	0.024

Age group: every ten years above 50 years old; ICU-6: 6 hours after intensive care unit admission; eRQ: estimated respiratory quotient; ICU-12: 12 hours after intensive care unit admission.

In the second model of analysis, "ICU-12 ΔPCO_2_" was included, and "ICU-12 eRQ" was excluded. Similar results were found for Euro SCORE, ICU-6 lactate and ICU-12 ΔPCO_2_, which were independently associated with complicated course ( Table 4 ).

**Table 4:** Logistic regression for predicting a complicated clinical course after coronary artery bypass surgery in patients with left ventricular dysfunction (second model – without T4 eRQ)

Variable	Univariate logistic regression			Multivariate logistic regression		
	Odds ratio	95% CI	Univariate p	Odds ratio	95% CI	Multivariate p
Age group (10 years)	2.13	0.75 to 6.05	0.154	-	-	-
Weight (kg)	0.94	0.88 to 1.01	0.114	-	-	-
EuroSCORE	2.19	1.32 to 3.63	0.002	2.51	1.58 to 3.97	<0.001
Lactate ICU-6 (mmol/l)	1.61	1.07 to 2.43	0.022	1.68	1.10 to 2.56	0.016
ΔCO_2_ICU-12 (mmHg)	1.47	1.10 to 1.95	0.009	1.33	1.05 to 1.68	0.015

Age group: every ten years above 50 years old, ICU-6: 6 hours after intensive care unit admission; ΔCO_2_: venoarterial CO_2_gradient; ICU-12: 12 hours after intensive care unit admission.

### Receiver operating characteristic curves (ROC curve)

The optimal cutoff values of Euro SCORE, "ICU-12 ΔPCO_2_"_,_"ICU-12 eRQ," and "ICU-6 Lactate" to identify patients with complicated postoperative outcomes were defined by analyzing ROC curves ( Table 5 ). The areas under the ROC curves of the variables were not significantly different.

**Table 5:** Areas of receiver operating characteristic curves to predict a complicated postoperative clinical course after coronary artery bypass surgery in patients with left ventricular dysfunction

Parameter	Area	95% CI	p	Cutoff	Sensitivity (%)	Specificity (%)
EURO SCORE	0.76	0.66 to 0.86	<0.0001	5	78	65
Lactate ICU-6 (mmol/l)	0.67	0.55 to 0.80	0.0033	4	49	84
eRQ ICU-12 (mmHg/mL/dL)	0.68	0.57 to 0.80	0.0010	1.65	54	78
ΔCO_2_ICU-12 (mmHg)	0.72	0.60 to 0.83	<0.0001	6.9	62	80

ICU-6: 6 hours after intensive care unit admission; ΔCO_2_: venoarterial CO_2_gradient; ICU-12: 12 hours after intensive care unit admission; CI: Confidence interval.

Euro SCORE ≥ 5 predicted a worse clinical course with a sensitivity of 78% and a specificity of 65%. An eRQ ≥ 1.65 mmHg/mL/dL 12 h after ICU admission predicted a complicated outcome with a sensitivity of 54% and a specificity of 78%. ICU-12 ΔPCO_2_≥ 6.9 mmHg also predicted a complicated course with a sensitivity of 62% and a specificity of 80%, and ICU-6 lactate ≥ 4 mmol/L predicted complications with a sensitivity of 49% and a specificity of 84% ( Table 5 ).

## Discussion

The purpose of the present study was to evaluate whether the perioperative markers of tissue hypoperfusion are predictive of adverse postoperative outcomes in patients with left ventricular dysfunction who underwent coronary artery bypass surgery. The main results indicated that Euro SCORE, 6-hour postoperative arterial lactate, 12-hour postoperative eRQ and ΔpCO_2_are independent predictors of prolonged ICU length of stay.

Patients with left ventricular dysfunction who underwent coronary artery bypass surgery usually present higher rates of complications,^13^as observed in our study, resulting in a prolonged ICU stay. The early prediction of complicated postoperative evolution may require special attention to minimize complications.

In our data, it was possible to identify that some biomarkers of tissue perfusion differed in the first and 12 hours in the group of patients who had a favorable course. Our data suggest that a 12-hour postoperative eRQ is a reliable marker of tissue oxygen disturbances related to postoperative complications. The RQ has been studied in different clinical settings to indicate tissue hypoxia and anaerobic metabolism.^5 , 7 , 16 - 18^Nevertheless, its role as an outcome marker is still not well defined. Mekontso-Dessap et al.^5^studied critically ill patients and suggested that an eRQ of higher than 1.4 mmHg/mL/dL estimates a lower overall survival at one month.^5^Lundin et al.^16^showed that 24 hours after cardiac arrest, levels of RQ were independently associated with ICU mortality but not with neurologic outcome three months later. In contrast, ΔpCO_2_was associated with a lower rate of ICU mortality and a poor neurologic outcome. Among lactate, ScvO_2_and ΔpCO_2_, only ΔpCO_2_was able to predict the worst outcome.^16^

The present study examined the predictive value of eRQ in high-risk cardiac surgery patients. Our findings suggest that an eRQ higher than 1.65 mmHg/mL/dL could predict postoperative complications, with 54% sensitivity and 78% specificity when analyzed 12 h after ICU admission.

A high RQ value is related to anaerobic metabolism when DO_2_is inadequate to meet O_2_demand (VO_2_). The physiological response to DO_2_decreases in the tissues and leads to an increase in the ERO_2_of capillary blood to maintain adenosine triphosphate (ATP) production and cellular energy demand. However, with critical decreases in DO_2_, compensatory increases in ERO_2_may not be sufficient to provide the O_2_required to sustain aerobic metabolism. In this setting, cellular VO_2_reductions cause anaerobic CO_2_production. This imbalance between oxygen supply and demand explains an increased RQ (VCO_2_/VO_2_) when DO_2_drops to critical levels.^17^

Elevated ΔpCO_2_analyzed at 12 hours after ICU admission and high arterial lactate concentration at 6 hours postoperatively were also associated with poor outcomes. The values of other global perfusion markers, BE, SvO_2,_C_a-v_O_2_, DO_2_, VO_2_, O_2_ER, and cardiac index, were not different.

The physiological mechanism of venoarterial pCO_2_difference (ΔPCO_2_) elevation seems to be more related to low blood flow states than to tissue hypoxia with anaerobic production of CO_2_. Vallet et al.^19^demonstrated in an experimental model of hypoxic and ischemic hypoxia that lowering DO_2_by decreasing blood flow resulted in an increased ΔPCO_2_, whereas lowering DO_2_by reducing blood oxygenation does not affect ΔPCO_2_.^19^The CO_2_stagnation phenomenon can explain the widening of ΔPCO2 in low blood flow states. The blood flow transit time slowed, resulting in an increased addition of CO_2_per unit of blood crossing the efferent microvessels and generating venous hypercarbia.^20^

A high value of venoarterial pCO_2_difference has been related to an unfavorable outcome in different clinical scenarios.^9 , 21^In cardiac surgery, Cavaliere et al.^9^observed an association between elevated ΔPCO_2_and postoperative complications. Difficulty weaning from CPB was also related to high values of ΔPCO_2,_as demonstrated by Denaut et al.^22^According to our results, a ΔPCO_2_higher than 6.9 mmHg correlates with a negative postoperative outcome. ROC curves presented 62% sensitivity and 80% specificity when analyzing ΔPCO_2_12 h after ICU admission.

Interestingly, initial values of ΔPCO_2_and eRQ at ICU admission and 6 hours after were not associated with outcomes in our patients. These findings suggest that the initial values of tissue perfusion parameters were not as predictive as later values when linked to clinical course. Inflammation in these patients, which was more pronounced in the first hours after CPB,^10^resulted in higher blood flows and could explain why eRQ was more related to ΔPCO_2_than to arterial lactate. Such elevation in eRQ may be explained by mathematical equation including ΔPCO_2_rather than by tissue hypoxia and anaerobic metabolism.

High levels of plasma lactate were also related to prolonged ICU stay in our study. Hyperlactatemia is a well-recognized marker of circulatory failure, and its values have been associated with increased mortality in various clinical settings.^3 , 8 , 23 , 24^During cardiac surgery with CPB, a high lactate concentration is frequently (10 to 20%) associated with postoperative morbidity and mortality.^3 , 25^The causes of hyperlactatemia during and after cardiac surgery remain controversial. Most authors attribute this finding to tissue hypoxia (type A hyperlactatemia). However, type B hyperlactatemia, which occurs in patients without tissue hypoxia, may occasionally be seen after CPB.^3 , 26 , 27^In this study, the arterial lactate concentration increased after surgery and remained elevated until ICU admission, when it decreased progressively. Six-hour postoperative lactate levels were significantly higher in the prolonged ICU stay group. However, when analyzed 12 hours after ICU admission, the lactate levels were normal. This difference was not maintained, suggesting that the lactate elevation was related to intraoperative tissue hypoperfusion, which contributed to unfavorable course in our population. According to our results, an arterial lactate concentration ≥ 4 mmol/L at ICU-6 predicted a complicated outcome with a sensitivity of 49% and a specificity of 84%. This lack of sensitivity was identified in a previous study^28^and suggests that initial lactate levels cannot predict certain postoperative adverse events.

Patients with unfavorable outcomes also presented a higher Euro SCORE value. The correlation between Euro SCORE and complicated evolution in the ICU has been described,^15 , 29^which emphasizes the importance of Euro SCORE as a screening parameter to predict ICU length of stay.

As demonstrated in previous studies,^30 , 31^oxygen-derived parameters are poorly correlated with anaerobic metabolism and therefore cannot be used as prognostic indicators in our specific population. The interpretation of a low VO_2_is difficult; it can be related to tissue hypoxia or a reduced O_2_demand without systemic hypoxia or low temperature. Low values of SvO_2_can be associated with global tissue hypoxia, an acute decrease in DO_2_, or with aerobic conditions if compensatory mechanisms of O_2_extraction were inadequate. On the other hand, average or even high values of SvO_2_can be associated with profound tissue hypoxia related to impaired O_2_extraction or low metabolism caused by deep anesthesia and hypothermia. An imbalance of temperature in different body compartments related to nonuniform bypass rewarming^32^may sometimes explain the high initial values of SvO_2._Similar conclusions can be drawn for O_2_ER and C_a-v_O_2_. Accordingly, we did not find significant differences between SvO_2_, DO_2_, VO_2_, O_2_ER, and C_a-v_O_2_.

### Limitations of this study

Limitations of our study should be acknowledged. The confounding factors may have influenced the results; for example, the inflammatory state that leads to abnormally high levels of cardiac output and inadequate hemodynamic optimization. Elevated postoperative eRQ, ΔPCO_2,_lactate, and higher EuroSCORE were significantly correlated with a complicated postoperative course. However, we cannot conclude whether such elevation in these hypoperfusion parameter was related to an insufficient optimization with vasoactive drugs or to refractoriness to treatment. Therefore, optimizing tissue perfusion in high-risk patients submitted to cardiac surgery should be further studied using optimal eRQ, ΔPCO_2,_and lactate values as targets.

## Conclusion

In conclusion, our findings have shown that Euro SCORE, arterial lactate at six hours postoperatively, ΔPCO^2^at 12 hours postoperatively, and eRQ are independent predictors of adverse outcomes in patients with left ventricular dysfunction after cardiac surgery. The predictive power of these hypoperfusion parameters is independent of the preoperative factors represented by the EuroSCORE. There was no superiority of any biomarker identified as an independent predictor.
